# P-697. Symptom Burden During the First Week of Acute Influenza Infection Among US Adults: An Interim Analysis of a Nationwide Prospective Study during the 2024/25 Season

**DOI:** 10.1093/ofid/ofaf695.910

**Published:** 2026-01-11

**Authors:** Tianyan Hu, Laura L Lupton, Alon Yehoshua, Joseph C Cappelleri, Meghan Gavaghan, Verna Welch, Santiago M C Lopez, Manuela Di Fusco, Xiaowu Sun

**Affiliations:** Pfizer Inc., New York, New York; CVS Health Clinical Trial Services, Woonsocket, Rhode Island; Pfizer Inc., New York, New York; Pfizer Inc., New York, New York; Pfizer Inc., New York, New York; Pfizer, Collegeville, Pennsylvania; Pfizer Inc, Collegeville, Pennsylvania; Pfizer Inc, Collegeville, Pennsylvania; CVS Health, Woonsocket, Rhode Island

## Abstract

**Background:**

This study described the frequency and severity of acute symptoms among outpatient adults with test-confirmed influenza infection in the US during the 2024/25 season.

**Methods:**

The study recruited symptomatic adults with test-confirmed influenza infection at CVS Health between 10/24/2024-4/15/2025 (CT.gov: NCT05160636). Socio-demographics, clinical characteristics, and vaccination status were collected at enrollment via an online survey. The frequency and severity of 12 acute symptoms were recalled for the pre-infection baseline at enrollment and followed up at Week 1. Severity was rated on a 4-point scale (0=none, 1=mild, 2=moderate, 3=severe). Descriptive statistics were used to summarize numbers of total symptoms and moderate/severe symptoms. Outcomes were compared between time points using paired t-tests.

**Results:**

Of 720 participants, mean age was 42.0 (SD: 13.0) with 70.6% between age 18-49 years old and 29.4% in ≥50 years old. Among all participants, 73.8% were female, 47.1% had ≥1 comorbidity, 84.4% self-reported antiviral use, and 48.7% received influenza vaccinations recommended for the 2024/25 respiratory season. The most frequently reported symptoms at Week 1 were cough (75.2%) and stuffy or runny nose (65.0%), followed by fatigue or tiredness (39.0%) and then post-exertional malaise (31.0%). These were also the most frequently reported moderate/severe symptoms at Week 1, with moderate/severe cough reported by 16.7%, and moderate/severe stuffy or runny nose reported by 10.0%. At Week 1, participants had a mean of 2.9 (SD: 2.2) acute symptoms, which was a statistically significant increase (+1.8; p< 0.001) relative to the pre-infection baseline.

**Conclusion:**

This study findings indicate that influenza-related respiratory symptoms were substantial and persisted one week after infection in the most recent season in the US, with cough and stuffy or running nose as the most frequently reported symptoms. These results highlight the need for continued monitoring of symptom burden over time and understanding of symptom progression. These data raise awareness of patient burden and underscore the importance of preventative measures such as influenza vaccination.

**Disclosures:**

Tianyan Hu, PhD, Pfizer: Salary|Pfizer: Stocks/Bonds (Public Company) Laura L. Lupton, MD, MHSA, CVS Health: Employee|CVS Health: Stocks/Bonds (Public Company) Alon Yehoshua, PharmD, MS, Pfizer Inc: Employee of Pfizer and may hold stock or stock options of Pfizer Joseph C. Cappelleri, PhD, Pfizer Inc.: Employee|Pfizer Inc.: Stocks/Bonds (Public Company) Meghan Gavaghan, MPH, Pfizer Inc.: Employee Verna Welch, PhD, MPH, Pfizer Inc.: Employee and may hold stocks Santiago M.C. Lopez, MD, Pfizer Inc.: Employee of Pfizer Inc. and may hold stock or stock options|Pfizer Inc.: Stocks/Bonds (Public Company) Manuela Di Fusco, PhD, Pfizer Inc: Employee of Pfizer and may hold stock or stock options of Pfizer Xiaowu Sun, PhD, CVS Health: Employee|CVS Health: Stocks/Bonds (Public Company)

